# The cost of unmodeled biological complexity in artificial neural networks

**DOI:** 10.1016/j.patter.2025.101343

**Published:** 2025-08-15

**Authors:** Antonio Bikić, Corinna Kaspar, Wolfram H.P. Pernice

**Affiliations:** 1 Department for Physics and Astronomy, Kirchhoff Institute for Physics, Heidelberg University, Heidelberg, Germany; 2 Department of Physics, Institute of Physics, University of Münster, Münster, Germany; 3 Department of Physics, CeNTech – Center for Nanotechnology, University of Münster, Münster, Germany

**Keywords:** artificial intelligence, artificial neuron, optimization, neuromorphic computing, artificial neural networks, ion channels, pragmatism, functionalism, randomness

## Abstract

We propose the theories of pragmatism and functionalism to differentiate between artificial neural networks (ANNs) and biological neural networks (BNNs). While ANNs emulate some cell structures and function approximation mechanisms, questions remain about their ability to emulate intelligent behavior observed in BNNs. We propose that relying solely on biological structures suitable for function approximation may overlook pivotal aspects of ANNs’ development, limiting their potential to emulate robust intelligence. Specifically, we investigate the role of ion channels in biological neurons and the randomness they introduce. This randomness seems to be vital for spike generation, although it is not directly related to function approximation. We conclude that structures, which do not directly contribute to function approximation, play a significant role in controlled activity, such as behavior, and should be integrated more into the controlled activity of artificial systems.

## The bigger picture

Artificial neural networks (ANNs) are powerful tools and a foundational building block in today’s AI. However, they are typically engineered to solve specific tasks like image recognition or natural language processing. In contrast, biological systems, particularly the human brain, exhibit a kind of intelligence that is far more task responsive, resilient, and capable of long-term learning. This perspective argues that some of these differences stem from aspects that are, by design, omitted in the modeling of many current AI systems: biological features such as intrinsic randomness and complex internal dynamics, which are often ignored because they seem irrelevant to task-specific optimization. However, overlooking these features may limit how robust and generalizable artificial systems can ultimately become.

By drawing on insights from neuroscience, biology, engineering, and philosophy, this perspective makes a case for rethinking which aspects of biology matter for building intelligent machines. It explores how components like ion channels and neuromorphic hardware challenge the standard blueprint of AI. Rather than simply mirroring nature, this perspective calls for a principled approach to identifying which biological structures are functionally significant and how they might be meaningfully adapted for technological use. Understanding these often-overlooked dimensions could guide the development of AI systems that are not only highly capable, resilient, and flexible but also more aligned with how real organisms learn, adapt, and persist.

## Teaser

This perspective argues that artificial neural networks (ANNs), while effective as function approximators, omit biologically relevant structures, such as ion channels, that introduce intrinsic randomness. Drawing on pragmatism and functionalism, Bikić, Kaspar, and Pernice propose that non-optimizing structures may be essential for developing robust intelligence and highlight the characteristics of current ANN architectures when compared to the biological complexity of real neurons.

## Introduction

When choosing the system level of an analysis in order to rebuild some of a system’s capabilities, it is vital to take into account all the components enabling the projected system level. In the case of the brain, possible levels of analysis are neural oscillations or the neuron. The level of abstraction currently used for artificial neural networks (ANNs) is the neuron. This is also called the neuron doctrine.^1^ Engineers utilize this abstraction level to assemble a number of artificial neurons (ANs) into an ANN for function approximation, which is typically associated with cognitive capacities like navigation, motor control, or classification. In the context of function approximation, the desired optimum varies: one optimum that the algorithm approximates could be described as approximating the balance between accuracy and false positives or the ratio between minimizing absolute and squared errors.

Interesting for our purpose, however, is the relation between the chosen level of analysis (neuron), the learning paradigm (function approximation, as this concept offers generalization, adaptability, and artificial representations), and the goal (balancing the optimum). One might ask, to what extent is there a difference in the ANN’s functionality if some of the underlying components of the biological neuron (BN) that led to the development of our chosen level of analysis are omitted? To be useful in the current learning paradigm, components of ANNs mainly have to respond to function approximation toward an optimum. If a structure found in a biological neural network (BNN) does not serve function approximation, it is clearly omitted from the beginning in the assembling of the ANN. One example of such a structure not being emulated as functions of ANNs is ion channels. These gating mechanisms seem to operate on a random basis and possess no significant memory capabilities.

There exist well-known simulation environments for compartmental modeling, like GENESIS^2^ and NEURON,^3^ that are also able to simulate ion channels. However, these simulation environments provide a platform to understand BNs in that they make them intelligible for humans through partly idealized and therefore understandable structures. This means that BNs reconstructed in that way share little to no resemblance with the ANs in engineering approaches aiming to generate functionalities like edge detection. ANNs must never be confused with models of BNNs in general. ANNs are inspired by neuroscientific models of BNNs, but ANNs do not have to be biologically justified.^4^

Our focus is on ANNs that do not simulate or help to understand BNNs but rather try to generate the behavior found in BNNs as their functionality. In particular, we aim to show the potential of currently unused biological components found in BNNs and their possible impact on the engineering counterpart, the ANN. Implementing the ascribed function of the abovementioned ion channels in ANNs could have much more impact, in particular with regard to their use of randomness as a source for generating functionality.

We introduce two conceptual tools from philosophy, namely functionalism and pragmatism, that show why there is a lack of an ion channel equivalent in the first place and make an argument for the implementation of structures like ion channels. We suggest that our current level of analysis, the neuron, is chosen because it is a better fit for function approximation than, for instance, oscillations or other structures. Such choices affect the functionality of systems.

In this paper, we address the conceptual gap between biological and artificial neural systems by focusing on the role of functional attributions, modeling constraints, and the interpretive frames that guide system design. We focus on the kinds of structures and capacities that are considered relevant for a system to be counted as intelligent, and how this varies between biological and artificial contexts. We first examine the kinds of functions that are modeled or omitted in current ANNs; second, we explore how pragmatist epistemology and system theory can help make sense of such modeling decisions; and third, we analyze how functionally successful structures may still leave out critical complexity. We aim to show that, despite surface-level task success, ANNs often rely on narrow and externally framed function definitions that obscure significant deviations from biological intelligence.

Starting with our chosen level of analysis, we state the following: ANs are modeled after BNs, as the latter play a significant role in the development of problem-solving behavior.^5^^,^^6^ Consequently, since models approximate reality and partially idealize it,^7^ ANs are not duplicates of BNs.^8^ However, this model character of ANs does not pose a major problem, as ANs are geared to realize a specific functionality that is observed as a behavior in BNs. ANs were invented primarily as a postulated hypothetical species of neurons that were completely defined *qua* computation, i.e., ANs, by definition, leave out the detailed biology of cells.^1^

ANs consist of abstracted and transformed structures that are based on structures of BNs. These structures are subsequently cast or reified into engineering components, rendering them tangible and operational. Assembling ANs into an ANN creates what is commonly defined as a function approximator.^9^ This physical arrangement of components is based on the formal abstraction of a model, but the ANN uses properties of the physical components to make the abstract simulations work. These formal abstractions or simulations are not contained in ANs or ANNs as levels of analysis or even causal structures.

Reification of neurons means assembling electronic components into a platform capable of emulating a specific functionality. In this case, the functionality is mirrored in the simulation of a BN in the form of a mathematical function. Such a reified neuron is what we call an AN. This process of reification is deliberately designed to incur losses, i.e., forfeiting some of the BN’s properties. In this sense, ANs usually omit biological structures that cannot be ascribed a utility in an approximation process toward an optimum.

This does not only apply to processes that go beyond the scope of the optimization scheme: a specific type of glia cell (astrocytes) is assumed to play a substantial role in the regulation of neuronal activity^10^^,^^11^^,^^12^ but was rarely found in neuronal simulators, let alone in ANNs. Such “cutoffs” frequently occur since neural simulation software is not enabled to provide a simulation of, in this case, astrocytes.^13^

As we mentioned earlier, the neuron is chosen to be the right level of analysis and not, for instance, oscillations. Oscillations are a phenomenon that involves populations of neurons, which is why they are above the single-neuron level of description. Networks imitating cognition would look and work differently with another selection of the basic abstract components and chosen hardware. This is because the hardware also determines how fast and in what circumstances machines can operate and therefore what functionality is possible.

ANNs are software, and software, in turn, is nothing but a very abstract form of hardware manipulation. With the concept of software, we invented a technique that facilitates hardware manipulation (e.g., transistors) in order to achieve (computational) goals without having to know how hardware actually works. Software makes it possible to talk about variables, lists, control flows, epochs, and formulas. This is a way that is far more familiar to human thinking than choosing the arrangement of myriads of transistors that fits a problem that we captured in a mathematical way. The concepts we use to adapt hardware to our problems are highly abstracted ways of telling the hardware what switching pattern (of transistors) should be implemented.

The number of ion channels can change over time, probably changing the amount of properties they influence in the whole system, but the defining structure of these channels stays the same. Due to the randomness in behavior and the lack of memory of these components, we suggest that the behavior of ion channels would have a different effect in ANNs than they have in BNNs. This would only be the case, however, if integrating structures that are not subject to approximation toward an optimum constitutes an integral part of developing robust functionality comparable to intelligent behavior.

In BNNs, ion channels are central to spike generation and contribute intrinsic randomness due to their stochastic behavior and thermal noise. While spiking neural networks (SNNs) and neuromorphic hardware,^14^ a type of non-conventional hardware that we will introduce later in more detail, aim to emulate such behavior, structures like ion channels in BNNs are not part of feedback loops optimized toward fixed goals. Their number can vary over time, and their stochastic behavior remains resistant to direct control. This contrasts with ANNs, where randomness, such as in dropout or weight initialization,^15^ is explicitly engineered, externally controlled, and optimized for performance. In this sense, randomness in ANNs is extrinsic and functionalized, whereas in BNNs, it is intrinsic and structural. Our suggestion is not merely that the behavior of ion channels differs in artificial settings but that incorporating non-optimizing structures may play an underappreciated role in achieving the robustness of intelligent systems.

Moreover, BNNs contain numerous components that are absent in current ANN designs, such as glial cells or metabolic coupling mechanisms. These components interact with ion channels in complex, dynamic ways that are poorly understood and largely unmodeled.^16^^,^^17^ Even if an ion channel can be mathematically simulated in hardware, its functional contribution depends on the systemic architecture in which it is embedded. The biological substrate (wetware) and the artificial substrate (hardware) differ not only in material composition but in thermodynamic properties, multi-scale interdependence, and regulatory complexity. As such, functional equivalence cannot be assumed on the basis of local structural mimicry alone. The behavior of an ion channel is not an isolated property but relational, emerging from its integration into a highly adaptive, noisy, and evolvable system.

In this perspective, we first show that the basis of a fair juxtaposition of ANs and BNs rests on two principles well studied in philosophy and dominant in engineering: pragmatism and functionalism. Consequently, in attempting to compare ANs and BNs, we define and juxtapose two distinct kinds of controlled activity (functionality and behavior) and compare these in terms of performance.

We focus on BNs and ion channels in particular and show their role in the generation of spikes, the encoding of information, and in intelligent behavior in general. We then spotlight the emulated nature of ANs, highlighting essential aspects of BNs needed for the construction of ANs in neuromorphic hardware. Further, we suggest that the machine paradigm as the leading building plan for ANs and ANNs might cause an adverse narrowing of the selection of the components necessary to enable robust functionality.

## Functionality and behavior as two different kinds of controlled system activity

Both ANNs and BNNs produce controlled system activity: the outcome of their operations is regulated such that certain variables (for instance, those of a cost function) are held constant. This controlled activity gives rise to observable behavior in BNNs and to functionality in ANNs. Functionality, as we use the term here, is always linked to behavior, insofar as it denotes the system’s ability to realize a capacity that approximates intelligent action.

One way to juxtapose ANNs and BNNs in terms of task solving is to ask, could ANNs solve the same tasks that are solved by BNNs? This shifts the focus from structural similarity to performance, in line with a tradition dating back to Turing’s seminal proposal^18^ to evaluate intelligence through indistinguishability in task execution rather than internal composition.

From this perspective, engineering does not need to replicate the structural features of BNNs. The only structures of interest are those that guarantee the functioning of the ANN relative to its specified goal. As soon as a working configuration for an ANN is found, the correct solution to the problem is also found, because the working configuration is useful. This instrumentalist framing resonates with a prominent epistemological tradition: pragmatism.^19^^,^^20^^,^^21^

This pragmatist perspective also provides an insightful approach to how to think about function attribution in ANN engineering. In computational theory, algorithms are formalized as procedures that govern transitions between discrete states, according to deterministic or probabilistic rules. These states are individuated not by their physical makeup, but by the roles they play within a system: their relations to inputs, outputs, and other internal states. In that sense, a state is not defined by the system’s physical activity (e.g., switching patterns of hardware) but by its function in the overall organization. This perspective aligns with functionalism in the philosophy of mind,^22^^,^^23^ which defines mental states in terms of their causal-functional roles, not their physical implementation.

Across different functions, a system may exhibit different functionalities. We understand functionality as observer relative: an ANN is classifying objects, performing calculations, or searching for patterns only insofar as its operation is interpreted in that way by an external observer. It does not have an observer-independent, intrinsic meaning that specifies what the system is doing. There are, of course, theories—such as Piccinini’s mechanistic account of physical computation—stating that certain physical systems objectively implement computations by virtue of their causal-mechanical structure.^24^ However, we do not take current ANNs to meet the criteria of such systems. Rather than instantiating computation in an objective, mechanistic sense, we consider *current* ANNs to be artifacts whose functionality is imposed by task framing and external interpretation.

Functionality in ANNs is governed by algorithms. Algorithms as instructions for action and programs as their formulations in a programming language differ profoundly from implemented programs. An implemented program is a form of hardware manipulation derived from the very abstract specification contained within the algorithm. Empirical studies^25^ have shown that even when the fetch-execute cycle (as in a video game, for example) is fully known—that is, the way a machine is instructed to operate—it remains impossible to understand a microprocessor using standard neurophysiological methods. This is because algorithms and programs are abstractions and therefore not objectively contained in a machine. A program can be understood as a theory^26^ that makes the inner workings of an artifact intelligible, but it is under no circumstances a structuring or governing force inside the machine. Algorithms and programs always start as abstractions that are important for explanation, construction, and maintenance of a system, but need to be aligned with real-world circumstances.

Behavior, on the other hand, is defined as measurable activities of a biological organism.^27^ These activities are subject to biological evolution driven by aimless processes. Behavioral structures can exist without any of these structures being understood: birds, for instance, famously do not care about ornithology. In ANNs, some inner workings, like the emergence of features in hidden layers, are also not understood, but these systems would not come into existence without those building them having a sound understanding of matrices, vectors, and the like. Crucially, biological organisms do not strive for preset optima but for adaptation to the environment, which in turn enables them to live according to their instincts.

In the context of controlled activity, intelligence is mentioned frequently and has unfortunately become quite an ambiguous notion. It is often associated with goal-directed activity and problem solving or the resolution of uncertainty toward solving a goal. For a more detailed analysis, see, for instance, Hochberg.^28^ Such definitions, however, may face problems when considering living beings like *Physarum polycephalum*.^29^^,^^30^ A unicellular organism, *Physarum polycephalum* is capable of exhibiting behavior that can be interpreted as “solving mazes” and “learning,” where it would be hard to argue that its problem awareness is comparable to a living being that we would consider intelligent.

For our purposes, we will define intelligence as a wide set of controlled activities that enable the systems equipped with intelligence to withstand, navigate, and take advantage of their environment. This may include problem-solving, abstract thinking, but also other capabilities. Here, the concept of an environment is not limited to physical space. For disembodied systems such as large language models, the environment may consist of sequences of linguistic inputs, interaction protocols, or even the distributional structure of token prediction tasks. Thus, rather than drawing a hard line between embodied and disembodied intelligence, we aim to consider how different systems encounter and manage the constraints and dynamics of their respective operational domains.

Robust intelligence includes strategies of dealing with noise in the presented data, unexpected events, a satisfactory success rate in system-preserving activity, and the treatment of mistakes. All of these properties are crucial for prevailing in different (real-world) environments. How these capabilities are realized is irrelevant, as long as the system can prevail. Serban,^31^ for instance, presents a good overview for robustness in image classification.

As noted earlier, pragmatist thinking may offer a crucial framework for understanding the conditions under which robust forms of artificial intelligence emerge. Pragmatism refers to a philosophical tradition (particularly a family of epistemological theories) that emphasizes the practical efficacy of concepts and the usefulness of theories as measured by their experiential consequences. These theories tend to de-emphasize accurate representation, propositional certainty, or foundational structures and instead prioritize adaptability, context sensitivity, and problem-solving capacity.

This kind of thinking remains—often tacitly—prevalent in engineering, and it has also been extensively studied and further developed in philosophy. Roughly speaking, pragmatism demands that facts make a causal difference in the real world.^32^^,^^33^ The “right” outcome, then, is the one that demonstrates practical efficacy and usefulness. This is reflected in the notion of operational coherence,^34^ where an activity succeeds because its components function together seamlessly.

In other words, the pragmatist maxim legitimizes any approach as true and justified so long as it yields useful effects. Take the selection of weights in an ANN: their specific values do not need to be understood as long as they allow the network to produce the desired output. Similarly, consider a person who sets their wristwatch 15 min ahead to avoid being late. The belief that the watch shows the correct time is clearly false, but this “false” belief produces the intended outcome of punctuality.

Such thinking naturally lends itself to functionalism: not only as a framework for understanding cognitive phenomena but also as a guide for building systems that exhibit the kind of controlled activity associated with mental capacities. Functionalism already underpins many approaches to ANNs, which are often taken to model mental systems via their functional organization.

On one level, the term “function” in the context of ANNs refers to the mathematical mapping from inputs to outputs. In practice, however, this mapping is rarely transparent or fully specifiable (see the next paragraph). On another level, the term denotes systemic capacities or dispositions: what a system can do rather than how it is materially constituted.

There is, however, a risk of conflating such functional attributions with teleological interpretations, especially when systems are described as if they possess internal goals. While ANNs can certainly be characterized in terms of what they are engineered to achieve, such descriptions reflect external intentions rather than intrinsic purposes. Framing them in terms of capacities helps to avoid misleading implications about objective or natural functions in the biological or evolutionary sense.

We can interpret intelligent systems taking the design stance,^35^ i.e., we assume that intelligent systems follow a purpose. This interpretation, however, does not purport inherent and objective teleological structures. What is even more interesting for us is another claim made in functionalism: multiple realizability but not medium independence.^36^ In terms of ANNs, this means that an activity (such as behavior or a function) observed in system A can also, in principle, be realized in system B (given the right structure). As we mentioned earlier, the notion of a function in an inanimate system is misguided. However, what can be said is the following: ANNs, *qua* switching patterns, transpose their capacities into functionality. This means an observer can state that the system as a whole exhibits the expected functionality. Functionality simply means that ideality (what one had in mind when building a system) and reality (what the system actually does) are congruent.

Subsequently, the combination of pragmatism and functionalism creates a similarity in performance between ANNs and BNNs: it does not matter what specific assumption, parameter, or substrate leads to a machine exhibiting a certain functionality. Since it is not mental states that matter but functionality, i.e., a working arrangement, pragmatism comes in handy. As a result, the combination of pragmatism and functionalism leads to a narrowing toward those pragmatist solutions that are capable of creating the desired substrate-independent functionality.

The theory of functionalism has its roots in automata theory (theoretical computer science), behaviorism (psychology), and positivism (philosophy). Behaviorism is relevant for understanding current artificial systems, even though these systems do not resemble the type of systems classical behaviorists had in mind; for a more nuanced discussion, see Buckner’s^37^ take on rational machines. As we mentioned earlier, biology defines behavior as measurable activities of a biological organism.^11^ More generally, positivism—deeply entangled with behaviorism—implies that there is nothing intelligible outside of what can be measured.^38^ Consequently, what is interesting is the observable behavior of an organism, not its subjective states. The organism may have subjective states like pain, luck, or the experience of colors, but these states are irrelevant since only measurable behavior counts (behaviorism). However, what should be pointed out is that functionalism purposefully departed from behaviorism in allowing for the attribution of inner mental states, such as pain, which mediate between observable inputs and outputs.

Take the example of the pigeon species *Columba livia*,^39^ capable of diagnosing breast cancer: these birds have been trained through conditioning (a behaviorist method) to perform this task. The birds have no concept of a diagnosis, breast cancer, or illnesses in general. Still, without understanding what they are actually doing, the birds, once conditioned by the experimenters to do so, exhibited a behavior (or data) that can be evaluated by external criteria. It does not matter what the birds think or feel (their subjective states) or what their conditioned behavior is actually about in terms of intentionality. What matters is that the exhibited behavior meets the experimenters’ standards.

The way we train ANNs is *not identical* to the conditioning process, but the key principles are similar. In the case of pigeons, scientists need to know something about the biology of these birds, how to extract information out of them, about their ability to recognize patterns, what they eat, how to give them positive feedback, and so on. In the same way, engineers know about the architecture of the ANN, how it is designed to learn rules, and how to conduct backpropagation. Ultimately, in both cases, the focus is on meeting benchmarks of performance and aligning themselves with these criteria.

Functionalism, as we use it here for our purposes, should be understood as a tool for interpreting certain processes in an original system (here, BNNs) as functions that can be realized in a target system independent of the system’s substrate (here, ANNs). In engineering, this process is commonly known as an emulation. Functionalist approaches guarantee a targeted interpretation of the functionality of an ANN, while the goal does not necessarily have to be understood by or contained in the executing system as some abstract concept (see the pigeon example).

To clarify the notion of emulation, consider the biologically realized process of flight: in a simulation,^40^^,^^41^ the primary goal is to understand this process. The simulation relies on a model that, in this case, makes sense of a biological phenomenon.^42^ In an emulation, the nature and composition of the components designated to realize a function are irrelevant as long as these components achieve the desired functionality. Again, the realization of functionality follows a pragmatist account: language, as we mentioned earlier, does not have to be understood, nor does heat have to be subjectively or phenomenally^43^^,^^44^ experienced, if the response of the system has the same effects as if language were understood or heat experienced. Hence, understanding or semantics can be omitted. The focus here, then, is on those aspects enabling functionality while leaving out concepts that do not seem to play a crucial role to meet benchmarks such as understanding and subjective experience (pragmatism). Still, the functionalist approach ensures that functions are found in the original system that can be emulated substrate independently in the target system.

Thus, an emulation is the product of a functionalist approach. It differs from a simulation in that it realizes a structure in the form of a function in a target system, leaving aside everything not necessary for this particular function, like the beat of a bird’s wings when emulating the capability of flight.

The focus here is on figuring out how to implement, for instance, flying, in principle, not to understand the biological process of flying.

The machine paradigm—interpreting living beings as machines—is the leading approach when explaining the neuronal behavior or creating models of ANNs. It is, however, also possible to interpret the cell as a complex system. The machine paradigm is, in general, convenient for describing a biological cell. However, it tautologically only brings into focus the parts that can be described in terms of a machine. There is, after all, reasonable doubt that the machine paradigm can capture all the aspects of a living cell, including motion,^45^ size,^46^ and other properties.^47^

We have good evidence, but we still do not know for a fact whether the features of the brain that are essential to cognition are those that can be captured through computational models and realized in non-living systems. Furthermore, the mere fact that traditional electronic computers are not engineered in such a way that their use causes changes in their material structure could be a solid explanation for why the plasticity exhibited by ANNs differs widely from brain plasticity.^1^

Identifying the machine paradigm with the real cell results in an unjustified and too-strong equation of biological neurons with ANs: creating functionality creates functionality, not behavior. The actual functionality of ANNs resembles behavior in that it produces, for instance, sound patterns that can be interpreted as meaningful language. It does not primarily matter whether ANNs ascribe some meaning or no meaning at all to these patterns. This is a result of focusing on the desired functionality by means of the pragmatic maxim: it is not necessarily useful for a machine to also understand the sound patterns as long as these patterns can, in turn, be interpreted by humans so that they are useful.

Implementation of the machine paradigm, then, comes at a certain cost: first, one cannot expect ANNs to have actual understanding,^48^ i.e., human understanding. Second, the machine paradigm tends to exclude features that do not amplify, for instance, the model-fitting process of an ANN.

In contrast, when comparing ANNs and BNNs as two forms of controlled activity, what is actually being compared is functionality and behavior. Functionality in ANNs is the attempt to approximate a function that is set as an optimum. Compared to an evolution that is driven by aimless processes^49^ that can improve fit to nature, the ANN’s way of functioning should be interpreted as goal directed without actually containing objective teleological structures.^8^ In biology, however, the view of teleological processes in living beings is often considered untenable.^50^ The need for such clarification arises because design and function are inherent to the engineering approach. In contrast, if a system is viewed as a physical system, i.e., a system of causal interactions, there is no room for functions. When we view the brain through an engineering lens, it is tempting to assume the possibility of reverse engineering. This might be seen as support for the assumption that areas of the brain can truly be *for* something. And, although this is a very particular perspective, viewing the brain that way, of course, helps when trying to understand it.^1^

Some scientists have proposed that ANNs can be used for explaining the capacities of BNNs.^51^^,^^52^ In this context, it is worth mentioning that natural selection is sometimes thought of as a process of approximating toward an optimum. Here, one has to acknowledge that there is still no predefined goal that natural selection is aiming to reach and the environment sets the “standards” of what is optimal in a particular situation. There is no overall goal that could be identified with an optimum. If it is not a working assumption like in the design stance, it also seems evident that evolution is not about perfection, which is often associated with an optimum. Evolved organisms incorporate plenty of structures that can be considered imperfect, like the recurrent laryngeal nerve in mammals that takes a “detour” to the larynx. Although there are scientists who developed theories that consider natural selection, with some caveats, to be an approximation of an optimum,^53^^,^^54^ we do not use these definitions in this paper. This is because such theories seem to imply structures in nature that may be explainable without having to invoke optima and approximation processes toward them but potentially evoke some metaphysical problems. Moreover, omitting optima leaves out the teleological burden of goal directedness of inanimate processes like natural selection or evolution in general.

For this reason, again, the viable alternative is pragmatism: it is a pragmatist approach to use the machine paradigm for describing living cells and deducting useful structures for emulations, even though it is clear that aimless evolutionary processes are probably not heading to an optimum state.^55^ Here, pragmatism can be used to construct both functioning machines and theories.

This means that the substantive product of ANNs—functionality—rests on design assumptions that yield controlled activity, constrained to structures that approximate some form of optimum. All components of ANN architecture (feature selection, interpretation of ANs as function approximators, etc.) are chosen with the aim of constructing systems that approximate optimal strategies for navigating their environments. As mentioned earlier, this environment may also take the form of a dialogue situation, as in the case of large language models.

The notion of optimization differs across learning paradigms: in supervised learning, the system minimizes a predefined loss function on labeled data; in reinforcement learning, it aims to maximize cumulative reward over time; and in unsupervised learning, the objective may involve, for instance, clustering or compression of structures without explicit external targets.^36^ Although these paradigms share an optimization framework in a broad sense, the epistemic and functional implications of optimum differ substantially.

Pragmatism and functionalism can serve as valuable lenses through which ANN paradigms can be analyzed and refined. A productive entry point for this analysis is the concept of function and its relation to intelligence.

As indicated earlier, a pragmatist perspective understands the meaning and success of a model not in terms of correspondence to the world but in terms of its practical utility and consequences. This implies that an ANN’s success (whether in supervised classification, reinforcement learning, or unsupervised feature extraction) is observer dependent and tightly coupled to human-defined goals and contexts. For example, a supervised network trained on a labeled dataset achieves accuracy only relative to a specific classification utility framed by the designer.^56^ Metrics like accuracy or reward are therefore not absolute indicators of intelligence but conventions that reflect chosen evaluative standards. This view challenges the conventional framing of function approximation architectures by highlighting that what is approximated is not mind-independent functions but utility-driven mappings. While this may not undermine system performance, it reshapes our expectations for ANN autonomy and generalizability.

Functionalism goes one step further by raising the question, what causal roles must a system instantiate to count as realizing a cognitive function like seeing? Convolutional neural networks (CNNs), for example, illustrate this point well. These systems process images and classify features through layered operations. But does this mean a CNN *sees*? Functionalism suggests that realizing a function like seeing requires a network of causal roles, often more extensive than what current CNNs instantiate. This highlights the problem of function individuation: it is often unclear what exact function a CNN is realizing (e.g., classification via low-level pixel correlations versus higher-order feature abstraction). Even in unsupervised learning, where the system discovers patterns autonomously, the functional frame (clustering, compression, and reconstruction) is still defined by the human designer. Functionalism thus emphasizes that identifying which causal roles matter depends on how function is construed. Pragmatism supplements this claim: what counts as a relevant causal role will shift depending on the system’s intended use and the context of deployment.

In the following, we show a structure present in BNNs but omitted in ANNs and suggest why overlooking such structures may lead to undesired outcomes over time.

## BNs and ion channels

**Figure 1:**
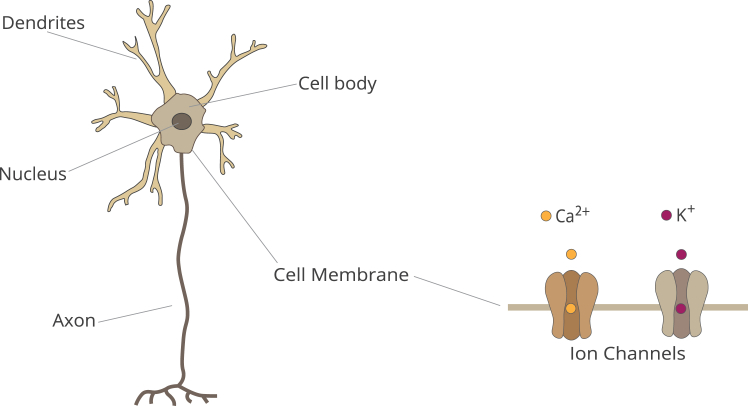
Illustration of a neuron with ion channels highlighted Left: a schematic representation of a neuron. Note that the cell body contains a diverse set of organelles, such as the Golgi apparatus and mitochondria. The latter, for instance, can also function as a mediator of signals. Right: two different ion channels shown in detail.

BNs (see Figure 1) form a complex network via numerous synapses. According to the current understanding of BNNs, action potentials, or so-called spikes, are the basis of communication between individual neurons. Action potentials also have an influence on the network’s architecture and, thus, are crucial for any cognitive inquiry. When a neuron fires, an action potential propagates along its axon toward the terminals, where it triggers the release of neurotransmitters carried by synaptic vesicles into the synaptic cleft.^57^ These neurotransmitters cross the synaptic cleft and elicit a specific signal in the post-synaptic neuron.^58^

This threshold-dependent firing is also a tool being used in ANNs that do not use rate models but work with artificial spikes. The underlying biophysical principle of an action potential is the temporary deviation of the neuron’s cell membrane potential from its resting value. A fundamental component in the process of action potential generation and, thus, of the principle functioning of BNNs is voltage-gated ion channels.^59^^,^^60^ These membrane-spanning proteins change their permeability for specific ions depending on the actual membrane potential. In this way, the proteins govern the ion flux across the membrane and thereby propagate the action potential. The channel’s sensitivity to the membrane voltage is due to a voltage-sensing domain. Charges inside this domain are translocated when the membrane potential changes. The consequence is a conformational change of the channel protein, which leads to the opening or closing of a pore through which specific ions can pass.^61^^,^^62^^,^^63^^,^^64^

It is obvious that the intricacy of a task, which a neural network is able to solve, scales with the complexity of the network’s architecture. The complexity of BNNs emerges from multiple connections between different neurons, each having a specific synaptic strength, but also from the individual structure of the involved neurons. In particular, there are many classes and subclasses of ion channels, which greatly differ in their properties, such as kinetics, activation threshold, or ion specificity.^65^ The variety of possible compositions and spatial distribution of ion channels expressed by a single neuron constitutes a high-dimensional parameter space. Therefore, each neuron individually responds to a defined input signal depending on its specific constitution. In a network consisting of multiple neurons, this complexity rises to an even greater magnitude.

Complexity is one of the key drivers of evolving intelligent systems. One way to show this is to compare the giant squid to human beings. An action potential in the giant squid axon is generated with only two basic groups of voltage-gated ion channels, whereas signal transduction in human neurons is grounded on numerous different ion channels and even subtypes of the same.^66^^,^^67^^,^^68^^,^^69^^,^^70^

This contrast illustrates the diversity of neuronal architectures across species, potentially reflecting adaptations to different ecological demands. While a direct link between ion channel heterogeneity and intelligence remains speculative, such complexity may contribute to the broader functional repertoire of the nervous system.

Current ANNs mostly communicate via synapses; structures such as ion channels are entirely omitted. At present, we cannot infer a causal relation between ion channels and intelligent behavior. Also, we are, for now, unable to simulate all of the components of a neuron, let alone a BNN, at once. However, the increasing differentiation of ion channels that a switch to a more complex biological niche entails seems to reflect a categorical change in the tasks that can be solved. Also, the ion channel’s ability for selective permeability of ions that influence spike generation and membrane potential, leading to the generation of spikes and spike patterns in general, at least indicates a strong correlation between these two structures.^71^

Furthermore, the voltage sensitivity of ion channels offers the possibility of being modulated by cell-external factors, such as the surrounding ion concentration, and even by neighboring neurons.^72^^,^^73^ This leads to additional communication pathways so that much more complex signal patterns can be created and a much more sophisticated system of information processing results.

The question is then, can an arbitrarily complex ANN based on function approximation comprehensively emulate biological intelligence? Even implementing ion channels in ANNs does not seem to allow an affirmative answer, since the randomness of ion channels would most likely contribute to an approximation process differently, which is the basic operation principle of ANNs.

Another question still unanswered in neuroscience is the encoding of information in spiking neurons, which can involve either the spike rate or the spike timing—or both.^74^^,^^75^^,^^76^ In contrast to ANs in common deep neural networks, current SNNs try to implement this time-dependent property, albeit in a very rudimentary way: the current membrane potential represents the neuron’s state, and only if a certain threshold is exceeded, resulting from time-dependent inputs, is a spike generated. However, it is still questionable if this representation comes any closer to intelligence made up by BNNs. Not only might the information encoding in spike rate and spike timing be of importance for intelligent behavior but also the corresponding sophisticated implementation in BNs, which, again, might not exclusively be geared toward approximation.

The activity of voltage-gated ion channels is often described by means of a Markov process. This means that the probability of a channel changing from “open” to “closed,” or vice versa, is independent of the channel’s history but only depends on the current state. Thus, the channel does not feature a memory and its activity is considered probabilistic.^77^ However, for a structure to be involved in an approximation process, it must be capable of processing feedback, for which memory is indispensable. Some models of ion channels that include memory fit well to experimental data of the opening/closing mechanisms of specific channels. The memory is, for example, explained with a coupled oscillator model: the charges in the voltage-sensing domain of the channel are influenced by both the structure of the protein molecule and the environment.^78^ Depending on the exact initial conditions, the voltage-sensing domain reacts to a change in transmembrane potential in a way that is predetermined by probabilities derived from “memorized” processes. However, the initial situation is not directly coupled to the preceding activity of the neuron, which is why the following activity is random. Therefore, there cannot be feedback, which would enable an approximation process toward a determined optimum.

One could argue that the feedback is not directed to the ion channel itself but rather intervenes in much more fundamental processes.^79^ A neuron that is completely isolated from external inputs and left to itself adapts mRNA levels within hours, which in turn determine the exact composition of expressed channel types in the cell membrane. This adaptation prevents the cell from bursting and mainly ensures its survival in a modified environment. The neuron does not optimize any criteria given to the network but rather comes up with a possible (not necessarily the optimal) solution to survive. In particular, there is no direct, action-related feedback to the ion channels in the cell membrane after their opening/closing activity.

## ANs

**Figure 2:**
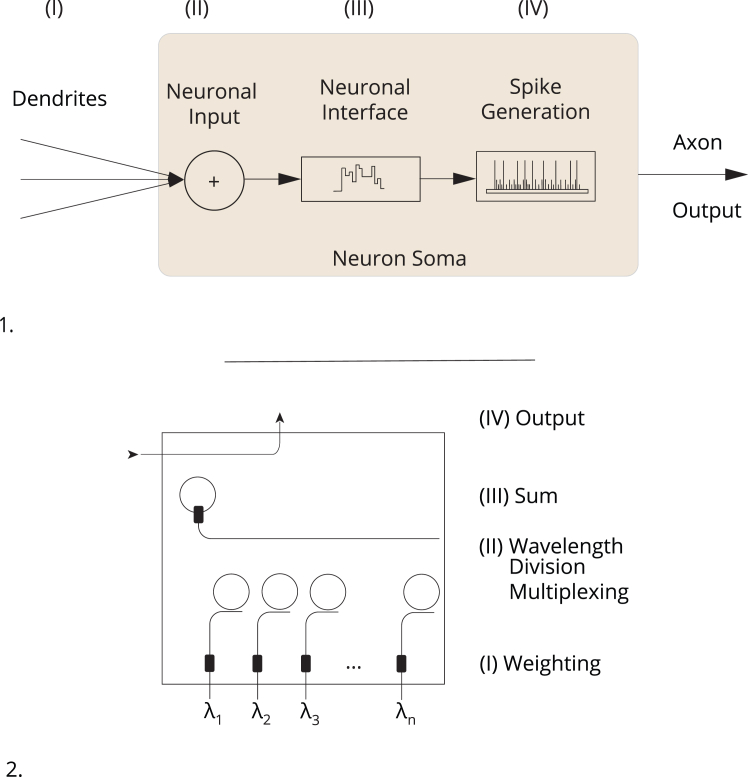
Artificial neurons (1) Neuromorphic circuit (schema). (2) Wiring diagram. This graphic shows how information encoded in spikes on different wavelengths of light (λ_1__–__n_) enter the optical circuit. In the first step, these spikes (input) are weighted through phase-change materials (PCMs). In a second step, the spikes are combined into a single waveguide using a wavelength division multiplexer (II). The wavelengths stay separate if they have different lengths and, unlike electrons, do not mix. In the third step, they are summed up (III). If the post-synaptic spikes surpass a certain threshold due to their summed up power, a PCM cell switches and generates an output (neuronal spike). For the original all-optical spiking neuronal circuit, see Feldmann.^81^ When juxtaposing a biological neuron (see Figure 1) and an artificial neuron based on neuromorphic hardware, one can clearly see the differences between these two action units. Not only are structures like ion channels or the nucleus left out in ANs, but every component that is being exploited is realized entirely differently. As a result, the best measure of comparison of these two action units is their performance. This performance, however, is exhibited through different processes, namely behavior (BN) and functionality (AN), meaning that these two action units feed from different sources of controlled activity.

We can legitimately investigate the similarities between biological neurons and ANs if we base this comparison on the theories of pragmatism and functionalism. Engineering approaches in the form of ANNs reduce biological complexity while maintaining the effects of behavior in the form of functionality (see the less complex AN in Figure 2 compared to the BN in Figure 1). However, this reduction in complexity leads to a *modus operandi* that significantly alters the scope of ANs relative to BNs. That said, the reduction of complexity is not to be confused with the program of reductionism: some areas of research on BNNs suggest that a reductionist link between brain and behavior might be unlikely.^80^ If this holds true, it might also indicate that hardware is unsuitable to produce functionality similar to behavior in a reductionist fashion.

### The emulated nature of ANs

So-called Von Neumann processors are called conventional computing devices: all of the computers we use in our everyday lives use processors of that kind. They are paradigmatic for the separation of memory and data processing/computation. Non-conventional computing devices typically explore architectures without this strict separation of memory and processing. Neuromorphic hardware, which implements neuromorphic computing, is a subset of non-conventional computing devices and facilitates the creation of energy-efficient and rapid hardware, overcoming the so-called Von Neumann bottleneck: the central processing unit of a conventional computer processing the data faster than they can be retrieved from the memory. Consequently, the processor is idle during retrieval, and the performance ultimately slows down.^82^

Moreover, Von Neumann computers are not designed to efficiently implement in-memory processing; parallel processing of data with techniques like multi-core CPUs is therefore only possible with limitations or additional hardware. This entails disadvantages such as high energy consumption and inefficiencies for massive parallelism compared to some non-conventional approaches. Neuromorphic hardware orients toward a brain-like processing of data. In that way, it overcomes difficulties with parallel data processing and removes the need to store data in locations where they are not actually computed. The result is energy-efficient and fast hardware. It is ideally suited for mobile and permanent use. Also, it handles data-intensive tasks without the need to communicate permanently with a data center.

Recent types of ANs based on Von Neumann architectures emulate the behavior of living neurons. In terms of ANNs, this means that only the structures that are pertinent to the purpose of function approximation are assembled into an ANN.^9^ As a result, emulating the simulation means that behavior is reconstructed through idealized structures and then facilitated to make up a new entity (like a robotic bird). The emulation now produces functionality, while the organism produces behavior. This takes place in a system using function approximation to ensure the robustness of said functionality that, in this case, aims to work well, also outside of computationally modeled worlds.

However, ANs as used in the Von Neumann architecture exist only *in abstracto* and, as abstract objects,^83^^,^^84^ have neither causal powers nor—in contrast to cells—an actual “urge” to survive. They do not exist as physical objects but rather represent models through which hardware can be controlled. Thus, hardware does not change, in that it takes the form of an AN that is discernible from other electronic components. Besides neuromorphic photonic chips, so-called memristive (memory resistor) devices have emerged as key components in a different approach to neuromorphic hardware.^85^^,^^86^^,^^87^ Memristive devices combine memory and processing by design. A memristive device can adjust its resistance based on past voltages or currents and thus store an internal state of flux/charge. This mechanism emulates the behavior of a biological synapse with built-in memory, nonlinearity, and multistability. Incorporating such memristive devices into neural networks yields dynamics more akin to cerebral processing than the static input-output mappings of conventional ANNs. Memristive neural models naturally exhibit so-called rich phenomena, like coexisting attractors and chaotic oscillations, due to the memristor’s inherent nonlinear memory properties.^88^ Rich phenomena are typically understood as complex and context sensitive, open to multiple interpretations, and resistant to epistemological reduction due to their tendency to exhibit emergent properties.^89^^,^^90^

For instance, one could think of a memristive Hopfield neural network (HNN) model using two memristive devices per AN: one device emulating a self-connection or autapse (autós + synapse) and the other device simulating an electromagnetic induction effect. Such an HNN can generate diversified “butterfly” attractors. By tuning the parameters of the first memristive device, this HNN transitions between Lorenz-like double-wing and four-wing chaotic patterns, while the second memristor controls the replication of these wings, enabling complex multi-butterfly (multi-scroll) attractors. The upshot of such memristive HNNs is that they offer a dynamic, history-sensitive, multistable alternative to static, task-bound ANNs. Instead of mapping inputs to outputs via training, these systems evolve internal dynamics that can reflect changing environments or internal structures that can be viewed as goal-like functionality.^88^

One of the key properties of a memristive device is its retention of a state. Thus, simply changing the initial charge of one memristor transposes the network into a different coexisting attractor. Such a transposition can occur when switching between a double-wing and a four-wing butterfly regime, demonstrating initial-condition-dependent multistability unavailable in ordinary Hopfield networks.^88^

Similarly, researchers have devised hidden multiwing memristive neural networks in which the memristor’s dynamics lead to chaotic attractors with multiple wings even though the system possesses several stable equilibria. These multiwing attractors are termed “hidden” because they do not originate from any unstable fixed point; instead, the memristor’s multilevel memory creates complex oscillatory states that coexist with stable rest states. Such hidden chaos exemplifies extraordinary multistability, leading to a single memristive network harboring many possible behaviors depending on its history. Such functionality cannot be anticipated by traditional ANNs that lack internal stateful elements.^91^

Likewise, in neuron models emulating organic cell structures to a higher degree, memristors enable significant dynamics: a memristive Hindmarsh-Rose (HR) neuron model under electromagnetic stimulation was shown to produce multi-scroll chaotic attractors whose shape and count are tunable via the memristor’s internal parameters and the intensity of the applied electromagnetic input. This HR model is designed with no fixed-point equilibrium, and its memristive inductive coupling yields hidden multi-scroll chaos with intricately structured scrolls. Interestingly, the parity (even or odd number) of scroll wings is set by the memristor’s characteristics, and the total number of scrolls grows with stronger external stimulation. In that way, a built-in memory device controls, in part, a single AN with enormous multistability and chaos. This leads effectively to a memory-dependent computational device where the neuron’s current functionality depends on the path it took through state space.^92^

Collectively, these advanced memristive models highlight how integrating memristive devices equips neuromorphic systems with qualitatively new dynamics: multiple coexisting attractors, tunable chaos, and state-dependent functionality. These dynamics offer different solutions to the emulation of behavior that exceed static function approximators. In contrast to a conventional ANN that converges to one fixed-point attractor, a memristive network can natively store and switch between many latent states or oscillatory patterns, leveraging physical memory effects for computing. This means that memristive neuromorphic hardware can achieve a level of adaptive, brain-like complexity beyond the reach of standard neuron models.^88^

As we indicated earlier, neuromorphic hardware initiates a shift^93^: ANs now are physically existing units instead of being driven on general-purpose hardware, as was previously the case. Through neuromorphic computing, the highly specialized tissue forms of biological organisms find, to some extent, morphological expression in the hugely differentiated hardware.^94^ Whether a biological component gets selected in order to be mirrored in the form of an electronic component depends on the biological component’s exploitability for optimization, which can indirectly include randomness. Again, the functionalist approach and the pragmatist maxim are essential: in-memory computation^81^^,^^95^ and energy efficiency^96^ are the desired functionalities in neuromorphic hardware, and the focus is to exploit only those effects that are necessary for optimization.

One of the research paradigms in (computational) neuroscience and cognitive science aims to computationally explain how the brain produces understanding through function approximation, whereas ANN research is, roughly speaking, concerned with the performance of capacities.^97^^,^^98^^,^^99^ Whether the latter amounts to intelligent behavior or understanding is more of a philosophical question and therefore not necessarily the aim of science and engineering. While explaining the brain with the function approximation paradigm produces remarkable results,^100^ building brain-like structures with the same paradigm focuses strictly on components and structures that can be described by the machine paradigm. However, the components in the examined living system that cannot be exploited for optimization still might influence the development of intelligent behavior.

Given how many biological components are left out during the emulation of an ANN, it is at least not unreasonable to assume that some of these omitted components are indeed significant for the successful working of the entire biological organism. That said, our level of analysis (the neuron) does not have to be the fundamental level or the one with the most potential for cognition. Given the amount of unsolved problems in neuroscience and the complex, multifaceted dependencies of the mind, brain, and behavior, it is premature to exclude so many components of the brain that are—at least—conducive to brain activity.^1^

One obvious objection is that all models contain idealizations and are always mere approximations to a presupposed much richer reality. While hardware and cellular organisms are two very different entities that realize controlled activity, when viewed as models, they both rely on difference-making for the target phenomenon.^101^ There are several definitions of difference-making. Strevens provides a definition of logical difference-making: which elements make a difference in knowledge (of the scientist)? Considering what we know about the correlation between ion channels (neuronal spikes) and cognition leading to intelligence, it is fair to connect ion channels to intelligent behavior. Diseases in ion channels resulting in an uncontrolled firing of neurons, so-called channelopathies, can cause some types of epilepsy.^102^^,^^103^ Now, there seems to be a connection between neuronal spikes and high-level behavior in humans, so this structure makes a difference for the target phenomenon (intelligent behavior). It should, then, be taken into account that there needs to be some structure in the target system (the ANN) that can influence the spikes in a manner comparable to ion channels.

As mentioned earlier, a BNN does not rely on an adequate explanation or model to exhibit proper behavior. A BNN works independently of our understanding of the BNN. Consequently, it can be described computationally and still have relevant components or structures that are not grasped by a computational description. A computational description, for instance, could therefore model the behavior of a living system by only capturing four of its five mechanisms. One could understand this model, see the original system working, and still be misguided about its actual working since the substantial fifth component is missing in the model.

Similarly, when an ANN is assembled and crucial components are missing, for instance, those for the right spiking patterns used by neurons, the artificial system is going to lack substantial functionalities. Given the possibility of functionalist analogs of ion channels, one could ask, is it possible to incorporate components in an ANN that introduce randomness into function approximation beyond our current controlled approaches but still contribute to a resilient functionality similar to intelligent behavior?

If so, the complexity of a BNN, which would include non-optimizing structures, would be, to some extent, subject to normative requirements, if the emulation of intelligent behavior is the objective. Especially, SNNs realized with neuromorphic hardware might profit from additional input from biological information processing so that they could exploit structures like ion channels for the development of robust functionality.^104^ Generally, SNNs require structures that are tailored to their mechanisms (such as their own initialization schemes), i.e., different structures than those that would be imposed by ANN architecture upon SNNs.^105^

In conclusion, ion channels play a fundamental role in the encoding of information and do not seem to be embedded in a function approximation process toward an optimum, in that they do not get any feedback for the adaptation of their stochastic performance.^106^

## The randomness we need

It has been shown that incorporating ion channel-like randomness into artificial networks acts as a regularizer, improving both learning performance and robustness. For instance, deliberately injecting intrinsic noise (mimicking ion channel fluctuations) into SNNs yields competitive accuracy while significantly increasing resilience to perturbations compared to noise-free models.^107^ Since optimizing system performance remains a central goal in engineering, randomness is not introduced arbitrarily; rather, the aim is to identify the *optimal levels* of internal noise. These levels can help prevent pathological synchrony and support stable coding in tightly coupled circuits.^108^

Leveraging phenomena such as stochastic resonance and always-on synaptic noise has enabled neuromorphic systems to achieve comparable accuracy with fewer neurons while maintaining greater tolerance to noisy input data.^109^^,^^110^ These findings suggest that emulating the stochastic behavior of ion channels *in silico* not only enhances biological plausibility but also contributes to improved adaptability, generalization, and functional efficiency in artificial neural systems.

As we mentioned earlier, the opening and closing times in ion channels occur randomly. This randomness is believed to introduce variability in the firing patterns of neurons. It is this variability that allows for processes like reliable exploration: randomly generated response options can be explored, which potentially leads to new solutions for existing problems. In mathematical terms, we would conclude that ion channels are one way to escape local minima.^111^^,^^112^^,^^113^

Such randomness could be beneficial for ANNs since it could make them robust to noise: slight changes in neuronal activity would pose no challenge because the overall functionality is maintained. However, it is not obvious how to implement the right amount and type of randomness at what time and logical level in the ANN. Until now, isolating the ion channel’s randomness as a function so that it could be implemented in another system remains unfeasible. This is mirrored by the fact that although we know randomness has some profound effects on the system, we cannot grasp the exact difference-making it introduces and what kind of manipulations of it as a function are legitimate.

The incorrect degree of randomness could make it difficult to foresee the functionality the system exhibits or to assume a clear optimum that the ANN tries to approximate. As we mentioned earlier, it is problematic to talk about goals in living beings because of the aimless evolutionary processes, which require a general rejection of any teleological view.

Brains might have a spectrum of states that they strive to attain, such as staying flexible and adaptable while at the same time being robust against real-world scenarios. Ion channel randomness could be beneficial for that process but would endanger the overall orientation toward an overall optimum.

It is clear that ion channels have an impact on BNNs, while they clearly do not directly contribute to the optimization for the best possible solution. If we interpret the brain’s processes as function approximation, we have to acknowledge that this kind of interpretation presupposes a variety of processes that are not directly geared to approximating an optimum. These processes are clearly difference-makers and should be identified as functions that form the prerequisites for our interpretation of function approximation in brains. This, then, applies *a fortiori* to ANNs. If we find a functional analog for ion channels that resonates with the algorithms governing ANNs, we might have found one of the right prerequisites for robust artificial intelligence.

## Summary and perspectives

In the first instance, ANNs and BNNs differ in that they produce two distinct kinds of controlled activity, namely functionality and behavior. Understanding ANNs based on pragmatism and functionalism makes it possible to compare these action units in terms of performance.

This performance is currently achieved by modeling BNNs as ANNs while exclusively focusing on the components that are exploitable for function approximation toward an optimum. The product of this process is emulation. This, by definition, disregards all those components that make no causal contribution to approximating the optimum. Hence, the approach clearly makes sense in terms of function approximation. However, as we have shown, the form of intelligence needed to persist outside of computationally modeled worlds might also rely on structures that do not directly benefit function-approximating structures.

BNs make use of many different kinds of ion channels to generate action potentials, which are the basis for communicating with connected neurons. Consequently, a more diverse set of ion channels could allow for the generation of more complex signal patterns. However, these ion channels are not included in a directed feedback loop that would “tell” them if their preceding activity was helpful in the pursuit of an optimum intended by the network. This is because a single ion channel does not have a memory but rather behaves in a probabilistic fashion. An implementation of ion channels as a prerequisite for the functioning of ANNs with the goal to increase the complexity of the network architecture would probably improve the network’s capability to solve sophisticated tasks via function approximation.

To this end, a functionalist analog of ion channels would have to be included in the flow of the optimization process. Optimization (or contributing to optimization), however, is not the “purpose” of ion channels in BNNs. An implementation according to their purpose in BNNs would be useless for ANNs—at least for state-of-the-art ANNs. This is due to the fact that we, so far, do not know how to make randomness not only a tool but also an intrinsic part of ANNs. In other words, we currently do not know how to display the randomness and potentially other properties as functions that we can subsequently implement substrate independently. As long as we have not yet comprehensively identified the activity of ion channels as a task or identified what other unknown structures fulfill in BNNs in terms of difference-making, it will be unlikely that a mere copy of their function-approximating activity would lead to the same results in ANNs.

Looking ahead, several strategies with a strong theoretical basis could inform the design of neural systems that go beyond standard function approximation. One option would be to draw on mechanistic modeling, which focuses on replicating internal causal structures rather than merely matching input-output behavior. As discussed earlier, the framework of functionalism helps identify the internal causal roles required to instantiate systems-level properties such as seeing. In artificial systems, these roles need not exactly replicate biological structures but could be functionally emulated through novel design strategies. Such roles are often overlooked, based on the assumption that ANNs can autonomously discover the causal structures needed to solve a given task, regardless of how those structures relate to the modeled system-level properties. This shift in focus (from task-driven mappings to causal-role implementation) opens the door to exploiting mechanisms like ion channel dynamics or synaptic plasticity without requiring actual biological replication.

Such an approach could also challenge the dominance of fixed, externally imposed cost functions. Instead, learning systems might be designed to co-evolve their objectives in response to internal states, uncertainty, or contextual shifts: what we refer to as goal plasticity.^114^ This idea, inspired by biological systems, promotes architectures in which learning emerges not from static mappings but from interaction-dynamic regimes capable of adapting priorities and functionality over time. Together, these directions sketch a pathway for ANN design that is more structurally expressive, biologically informed, and responsive to complexity than current function approximation-based paradigms allow.

## Acknowledgments

We thank the anonymous reviewers for their detailed feedback, which helped refine our arguments. We are also grateful to Ravi Pradip for his help with the all-optical spiking neuronal circuit, to Nora Angleys for proofreading this perspective, and to Willem Rabe for assistance with the graphics. We also acknowledge funding from CRC 1459 Intelligent Matter (10.13039/501100001659DFG project number 433682494), the 10.13039/501100004869University of Münster, and the 10.13039/501100002347Federal Ministry of Education and Research (BMBF), as well as the Ministry of Science, Research and the Arts of Baden-Württemberg, within the framework of the Excellence Strategy of the Federal and State Governments of Germany.

## Declaration of interests

The authors declare no competing interests.
